# Essential Oil Compositions of Three Invasive *Conyza* Species Collected in Vietnam and Their Larvicidal Activities against *Aedes aegypti*, *Aedes albopictus*, and *Culex quinquefasciatus*

**DOI:** 10.3390/molecules25194576

**Published:** 2020-10-07

**Authors:** Tran Minh Hoi, Le Thi Huong, Hoang Van Chinh, Dang Viet Hau, Prabodh Satyal, Thieu Anh Tai, Do Ngoc Dai, Nguyen Huy Hung, Vu Thi Hien, William N Setzer

**Affiliations:** 1 Department of Plant Resources, Institute of Ecology and Biological Resources, Vietnam Academy of Science and Technology, Hanoi 100000, Vietnam; tranhoiiebr@gmail.com; 2 School of Natural Science Education, Vinh University, 182 Le Duan, Vinh City 43000, Vietnam; lehuong223@gmail.com; 3 Faculty of Natural Sciences, Hong Duc University, 365 Quang Trung, Thanh Hoa 440000, Vietnam; hoangvanchinh@hdu.edu.vn; 4 Center for Research and Technology Transfer, Vietnam Academy of Science and Technology, Hanoi 100000, Vietnam; hauhoahock20@gmail.com; 5 Aromatic Plant Research Center, 230 N 1200 E, Suite 102, Lehi, UT 84043, USA; psatyal@aromaticplant.org; 6 Department of Pharmacy, Duy Tan University, 03 Quang Trung, Da Nang 550000, Vietnam; anhtai0808qn@gmail.com; 7 Graduate University of Science and Technology, Vietnam Academy of Science and Technology, 18-Hoang Quoc Viet, Cau Giay, Hanoi 100000, Vietnam; daidn23@gmail.com; 8 Faculty of Agriculture, Forestry and Fishery, Nghe An College of Economics, 51-Ly Tu Trong, Vinh City 460000, Vietnam; 9 Center for Advanced Chemistry, Institute of Research and Development, Duy Tan University, 03 Quang Trung, Da Nang 550000, Vietnam; 10 Faculty of Hydrometerology, Ho Chi Minh City University of Natural Resources and Environment, Ho Chi Minh City 70000, Vietnam; hiensphoa@gmail.com; 11 Department of Chemistry, University of Alabama in Huntsville, Huntsville, AL 35899, USA

**Keywords:** *Erigeron*, *Conyza bonariensis*, *Conyza canadensis*, *Conyza sumatrensis*, mosquito, vector control

## Abstract

Mosquito-borne infectious diseases are a persistent problem in tropical regions of the world, including Southeast Asia. Vector control has relied principally on synthetic insecticides, but these have detrimental environmental effects and there is an increasing demand for plant-based agents to control insect pests. Invasive weedy plant species may be able to serve as readily available sources of essential oils, some of which may be useful as larvicidal agents for control of mosquito populations. We hypothesize that members of the genus *Conyza* (Asteraceae) may produce essential oils that may have mosquito larvicidal properties. The essential oils from the aerial parts of *Conyza bonariensis*, *C. canadensis*, and *C. sumatrensis* were obtained by hydrodistillation, analyzed by gas chromatography–mass spectrometry, and screened for mosquito larvicidal activity against *Aedes aegypti*, *Ae. albopictus* and *Culex quinquefasciatus*. The essential oils of *C. canadensis* and *C. sumatrensis*, both rich in limonene (41.5% and 25.5%, respectively), showed notable larvicidal activities against *Ae. aegypti* (24-h LC_50_ = 9.80 and 21.7 μg/mL, respectively) and *Ae. albopictus* (24-h LC_50_ = 18.0 and 19.1 μg/mL, respectively). These two *Conyza* species may, therefore, serve as sources for alternative, environmentally-benign larvicidal control agents.

## 1. Introduction

Mosquito-borne infectious diseases have been a continuous health problem in Southeast Asia, including Vietnam. Dengue fever and dengue hemorrhagic fever are particularly problematic and chikungunya fever is an emerging threat in the country [1,2]. *Aedes aegypti* (L.) (Diptera: Culicidae), the yellow fever mosquito, is a recognized vector of dengue fever virus, chikungunya fever virus, Zika virus, and yellow fever virus [3]. *Aedes albopictus* (Skuse) (Diptera: Culicidae), the Asian tiger mosquito, is a key vector of several pathogenic viruses, including yellow fever virus [4], dengue fever virus [5], chikungunya virus [6], and possibly Zika virus [7]. *Culex quinquefasciatus* Say (Diptera: Culicidae), the southern house mosquito, is a vector of lymphatic filariasis [8] as well as several arboviruses such as West Nile virus and St. Louis encephalitis virus [9] and possibly Zika virus [10].

Several members of the genus *Conyza* Less. (Asteraceae) have been introduced throughout the tropics and subtropics where they have become invasive weeds [11,12,13]. *Conyza bonariensis* (L.) Cronquist (syn. *Erigeron bonariensis* L.), flaxleaf fleabane, probably originated in South America [14], but has been introduced throughout Asia, Africa, Mexico and the southern United States, Europe, and Oceania [13,15]. *Conyza canadensis* (L.) Cronquist (syn. *Erigeron canadensis* L.), Canada fleabane, is native to North America, but is also now naturalized throughout Europe, Asia, and Oceania [13]. *Conyza sumatrensis* (Retz.) E. Walker (syn. *Erigeron sumatrensis* Retz.) is probably native to South America, but this species has also been naturalized in tropical and subtropical regions [16].

Non-native invasive plant species are generally detrimental to the local environments where they have been introduced. They can outcompete native plant species and reduce biodiversity [17], they can alter ecosystem functions [18], and can have substantial economic impacts [19]. Control methods for invasive plants have generally included application of herbicides, physical cutting, or burning [20]. However, harvesting invasive species for beneficial uses as a method for control of invasive species may provide economic incentives to offset eradication costs [21]. For example, *Melaleuca quinquinervia* trees in south Florida have been cut and chipped for landscape mulch and boiler fuel [22]; it has been suggested that mechanical harvesting of invasive cattail (*Typha* spp.), common reed (*Phragmites australis*), and reed canary grass (*Phalaris arundinacea*) from coastal wetlands of Lake Ontario can be used as an agricultural nutrient source or as a biofuel [23]. The leaf essential oil of *Solidago canadensis*, an invasive plant in Europe, has been evaluated as a potential insecticide and demonstrated moderate larvicidal activity against *Cx. quinquefasciatus* [24].

The use of synthetic pesticides for mosquito control has had detrimental effects on the environment [25,26]. They tend to be persistent, toxic to non-target organisms, and insecticide resistance has been steadily increasing in mosquito species [27]. Essential oils have been suggested as viable, environmentally benign, and renewable alternatives to synthetic pesticides [28,29,30,31,32]. We have recently studied several introduced invasive plant species in Vietnam for potential use as mosquito vector control agents [33,34,35], and as part of our ongoing efforts in identifying readily-available essential oils for mosquito control, we have examined three *Conyza* species for larvicidal activity against *Aedes aegypti*, *Aedes albopictus*, and *Culex quinquefasciatus*, with the aim of identifying new mosquito-control essential oils and the components responsible for the activity. 

## 2. Results and Discussion

### 2.1. Essential Oil Compositions

The essential oils from the aerial parts of *C. bonariensis*, *C. canadensis*, and *C. sumatrensis* were obtained by hydrodistillation in 1.10%, 1.37%, and 1.21% yield. The chemical compositions of the *Conyza* essential oils, determined using gas chromatography–mass spectrometry, are summarized in Table 1. *Conyza bonariensis* essential oil was dominated by sesquiterpenoids, especially *allo*-aromadendrene (41.2%), β-caryophyllene (13.3%), and caryophyllene oxide (12.2%). Concentrations of monoterpenoids (1.8%) and diterpenoids (trace) were relatively small. The essential oils of *C. canadensis* and *C. sumatrensis*, on the other hand, were rich in limonene (41.5% and 25.5%, respectively). The aerial parts essential oil of *C. sumatrensis* also had a large concentration of (*Z*)-lachnophyllum ester (20.7%). There is wide variation in the essential oil compositions of *Conyza* species, both between species and within the same species (see Table 2). This is not surprising given the very different geographical locations of the collection sites for these samples.

### 2.2. Mosquito Larvicidal Activity

The mosquito larvicidal activities of the *Conyza* essential oils are summarized in Table 3. The essential oil of *C. canadensis* showed the best larvicidal activity against both *Ae. aegypti* (24-h LC_50_ = 9.80 μg/mL) and *Ae. albopictus* (24-h LC_50_ = 18.0 μg/mL) and good larvicidal activity against *Cx. quinquefasciatus* (24-h LC_50_ = 39.4 μg/mL). *Conyza sumatrensis* essential oil also showed good larvicidal activity against the three mosquito species (24-h LC_50_ = 21.7, 19.1, and 26.7 μg/mL, respectively, for *Ae. aegypti*, *Ae. albopictus*, and *Cx. quinquefasciatus*). *Conyza bonariensis* essential oil was less active (24-h LC_50_ = 69.7, 81.1 and 130.0 μg/mL against *Ae. aegypti*, *Ae. albopictus*, and *Cx. quinquefasciatus*, respectively).

**Figure 1:**
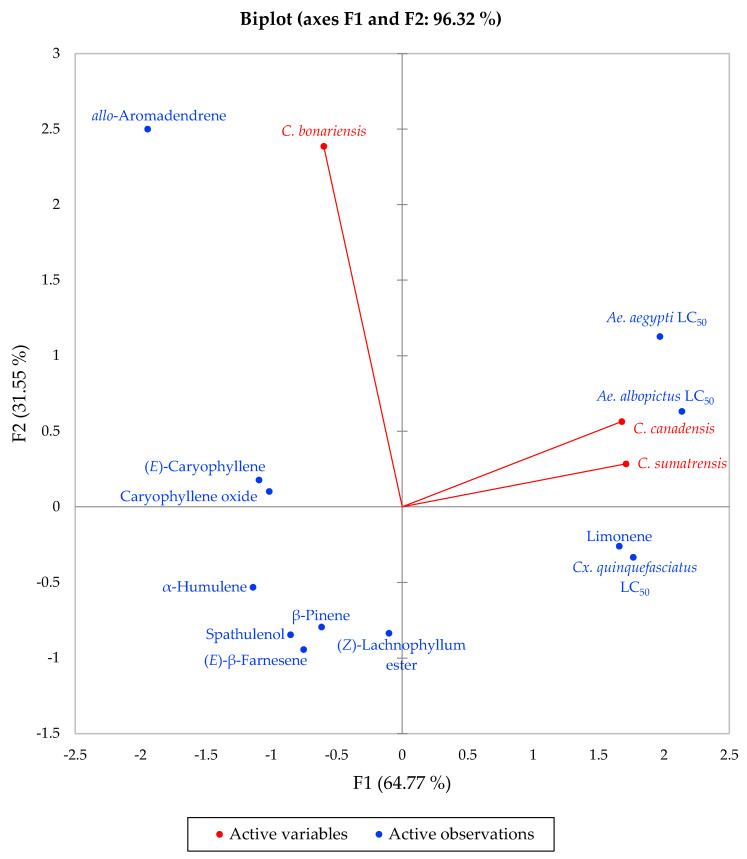
Principal component biplot of PC1 and PC2 scores and loadings demonstrating the relationships between *Conyza* essential oil major components and larvicidal activities.

The larvicidal activities of *Conyza* essential oils roughly coincides with the concentration of limonene in the samples (41.5%, 25.5%, and 0.2%, respectively, for *C. canadensis*, *C. sumatrensis*, and *C. bonariensis*), and this relationship is borne out in a principle component analysis based on the major essential oil components (limonene, *allo*-aromadendrene, (*Z*)-lachnophyllum ester, caryophyllene oxide, β-caryophyllene, β-pinene, (*E*)-β-farnesene, spathulenol, and α-humulene, along with the 24-h larvicidal activities) (Figure 1). Limonene has shown excellent larvicidal activities against *Ae. aegypti* (24-h LC_50_ = 17.7 μg/mL) and *Cx. quinquefasciatus* (24-h LC_50_ = 31.6 μg/mL) (Table 3) as well as *Ae. albopictus* (LC_50_ 10.8-41.8 μg/mL) [34]. Consistent with these results, Zeng and co-workers found the larvicidal activity of *C. canadensis* from China (14.8% limonene) to be 56.9 μg/mL and 32.1 μg/mL against *Ae. albopictus* and *Cx. quinquefasciatus*, respectively [54]. These workers also appreciated the remarkable larvicidal activity and noted that *C. canadensis* essential oil has a potential for further development. Furthermore, *Citrus* peel oils, rich in limonene, have also shown remarkable larvicidal activities against *Ae. albopictus* [61] and *Cx. quinquefasciatus* [62].

Other components in the *Conyza* essential oils likely contribute to the mosquito larvicidal effects. *Conyza bonariensis* was rich in (*E*)-caryophyllene (13.3%) and caryophyllene oxide (12.2%), but both of these compounds have been found to have weak larvicidal activities against *Ae. aegypti* (24-h LC_50_ = 70.8 and 137 μg/mL, respectively (Table 3). On the other hand, β-pinene, a major component of *C. canadensis* essential oil (8.8%), has shown larvicidal activity against *Ae. aegypti* (24-h LC_50_ = 23.6 μg/mL), *Cx. quinquefasciatus* (24-h LC_50_ = 30.5 μg/mL) (Table 3), and *Ae. albopictus* [61]. In addition, synergy between essential oil components may also be important [63,64]. Scalerandi and coworkers have found that the housefly (*Musca domestica*) metabolizes the major components in an essential oil, but leaves the minor components to act as toxicants [65].

In order to assess the potential detrimental impact of the *Conyza* essential oils on beneficial aquatic species, the insecticidal activity was assessed against the water bug, *Diplonychus rusticus*, an insect predator of mosquito larvae [66]. Both *C. canadensis* and *C. sumatrensis* essential oils were substantially less toxic to *D. rusticus* than they were to the mosquito larvae.

## 3. Materials and Methods

### 3.1. Chemicals

Chemicals used for this study, dimethylsulfoxide (DMSO), β-pinene, limonene, (*E*)-caryophyllene, α-humulene, caryophyllene oxide, dichloromethane, and permethrin, were obtained from Sigma-Aldrich (St. Louis, MO, USA) and used as received without further purification.

### 3.2. Plant Material

The three *Conyza* species were collected from Bach Ma National Park, Thue Thien Hue province (16° 11′ 34″ N, 107° 51′ 12″ E) in April 2020. The plants were identified by Dr. Do Ngoc Dai and Dr. Le Thi Huong. Voucher specimens, LTH129 (*Conyza canadensis*), LTH130 (*Conyza sumatrensis*), and LTH131 (*Conyza bonariensis*) have been deposited in the Pedagogical Institute of Science, Vinh University. Four-kg samples of fresh aerial parts (leaves, stems, and flowers) of each of the plants were shredded and hydrodistilled for 4 h using a Clevenger-type apparatus.

### 3.3. Gas Chromatography–Mass Spectrometry

The *Conyza* essential oils were analyzed by GC-MS as previously described [67]: Shimadzu GCMS-QP2010 Ultra, electron impact (EI) mode, electron energy = 70 eV, scan range = 40–400 atomic mass units, scan rate = 3.0 scans/s, ZB-5 fused silica capillary column (30 m × 0.25 mm, 0.25 μm film thickness), He carrier gas, 552 kPa column head pressure, and 1.37 mL/min flow rate. Injector temperature was 250 °C and the ion source temperature was 200 °C. The GC oven temperature program was programmed for 50 °C initial temperature, temperature increased at a rate of 2 °C/min to 260 °C. A 5% *w*/*v* solution of the sample in CH_2_Cl_2_ was prepared and 0.1 μL was injected with a splitting mode (30:1). Identification of the oil components was based on their retention indices determined by reference to a homologous series of *n*-alkanes, and by comparison of their mass spectral fragmentation patterns with those reported in the databases [36,37,38,39].

### 3.4. Mosquito Larvicidal Assay

Mosquito larvicidal activity was carried out on *Ae. aegypti*, *Ae. albopictus*, and *Cx. quinquefasciatus* as previously described [67]: For the assay, 1% stock solutions of each essential oil in dimethylsulfoxide (DMSO) were prepared, and aliquots of the stock solutions were placed in 500-mL beakers and added to water that contained 20 larvae (fourth instar). With each experiment, a set of controls using DMSO was also run for comparison. Mortality was recorded after 24 h and again after 48 h of exposure during which no nutritional supplement was added. The experiments were carried out 25 ± 2°C. Each test was conducted with four replicates with three concentrations (50, 25, and 12.5, μg/mL for *C. canadensis* and *C. sumatrensis*; 150, 100, and 50 μg/mL for *C. bonariensis*). Permethrin was used as a positive control.

### 3.5. Non-Target Insecticidal Assay

The *Diplonychus rusticus* adults were collected in the field and maintained in glass tanks (60 cm long × 50 cm wide) containing water at 25 °C with a water depth of 20 cm. The essential oils were tested at concentrations of 200, 150, 100, 75, 50, and 25 μg/mL. Four replicates were performed for each concentration. Twenty *D. rusticus* adults were introduced into each solution. The non-target organism was observed for mortality after 24 h and 48 h exposure.

### 3.6. Data Analysis

The mortalities were recorded 24 h and 48 h after treatment. The data obtained were subjected to log-probit analysis [68] to obtain LC_50_ values, LC_90_ values, 95% confidence limits, and chi square values using Minitab^®^ 18 (Minitab Inc., State College, PA, USA). For the principal component analysis (PCA), the 9 major components (limonene, *allo*-aromadendrene, (*Z*)-lachnophyllum ester, caryophyllene oxide, (*E*)-caryophyllene, β-pinene, (*E*)-β-farnesene, spathulenol, and α-humulene), and the 24-h larvicidal activities against *Ae. aegypti*, *Ae. albopictus*, and *Cx. quinquefasciatus* were taken as variables using a Pearson correlation matrix using XLSTAT Premium, version 2018.5 (Addinsoft, Paris, France). A total of 33 data (11 variables × 3 samples) were used for the PCA.

## 4. Conclusions

Invasive plant species are generally considered to be ecologically and detrimental with potential economic impacts, and the control or eradication of invasive plant species can be prohibitively costly. However, identification of beneficial uses of invasive plants could be economically advantageous and aid in the control of the species. *Conyza* spp., as well as *Erechtites* spp. [34], *Crassocephalum crepidioides* [35], and *Severinia monophylla* [33], are invasive weeds in Vietnam, and essential oils from these plants have demonstrated promising mosquito larvicidal activities. The plant materials are readily available and harvesting of these weeds may provide economically valuable “cash crops” as well as serve as a means for ecological remediation. Note that *C. bonariensis* [69], *C. canadensis* [70], and *C. sumatrensis* [71] have all shown resistance to the commonly used herbicide glyphosate, so herbicidal control of these weeds is impractical as well as environmentally detrimental. Further research on potential formulations (e.g., nanoemulsions or essential oil-loaded nanoparticles) [72] for field use of these promising essential oils is warranted. 

## Figures and Tables

**Table 1:** Chemical compositions of the aerial parts essential oils of *Conyza bonariensis*, *Conyza canadensis*, and *Conyza sumatrensis* collected in Vietnam.

RI_calc_^a^	RI_db_^b^	Compound	Relative Content %		
			*C. bonariensis*	*C. canadensis*	*C. sumatrensis*
931	932	α-Pinene	0.5	0.5	0.2
948	950	Camphene	tr ^c^	---	---
967	972	(3*Z*)-Octen-2-ol	---	---	tr
971	972	Sabinene	tr	0.1	0.1
976	978	β-Pinene	0.8	8.8	3.0
982	984	6-Methylhept-5-en-2-one	---	---	tr
987	989	Myrcene	tr	1.2	1.0
1023	1025	*p*-Cymene	tr	0.3	0.1
1028	1030	Limonene	0.2	41.5	25.5
1030	1031	β-Phellandrene	---	tr	---
1034	1034	(*Z*)-β-Ocimene	---	---	tr
1044	1045	(*E*)-β-Ocimene	---	tr	1.9
1049	1051	2,3,6-Trimethylhepta-1,5-diene	---	tr	---
1056	1057	γ-Terpinene	---	tr	---
1088	1091	*p*-Cymenene	---	0.1	---
1090	1091	Rosefuran	---	---	0.1
1093	1097	α-Pinene oxide	---	---	0.2
1097	1098	Perillene	---	0.1	---
1098	1101	Linalool	0.2	---	---
1101	1101	6-Methyl-3,5-heptadien-2-one	---	---	0.1
1103	1104	Nonanal	tr	---	---
1112	1113	4,8-Dimethylnona-1,3,7-triene	---	---	0.2
1118	1119	*endo*-Fenchol	tr	---	---
1120	1121	*trans*-*p*-Mentha-2,8-dien-1-ol	---	0.9	0.2
1124	1131	Cyclooctanone	---	0.8	---
1129	1130	4-Acetyl-1-methylcyclohexene	---	0.1	---
1131	1132	*cis*-Limonene oxide	---	0.6	0.2
1134	1137	*cis*-*p*-Mentha-2,8-dien-1-ol	---	1.2	0.3
1135	1137	*trans*-Limonene oxide	---	0.6	---
1137	1137	Nopinone	---	0.4	---
1137	1139	(*E*)-Myroxide	---	---	0.1
1139	1141	*trans*-Pinocarveol	tr	1.6	0.1
1150	1152	Citronellal	---	0.1	---
1160	1164	Pinocarvone	---	0.8	tr
1170	1170	Borneol	tr	---	---
1177	1179	2-Isopropenyl-5-methylhex-4-enal	---	0.3	---
1182	1184	*p*-Methylacetophenone	---	0.3	---
1185	1185	Cryptone	---	0.4	---
1185	1187	*trans*-*p*-Mentha-1(7),8-dien-2-ol	---	0.2	---
1189	1190	Methyl salicylate	tr	---	---
1193	1195	α-Terpineol	0.1	---	0.1
1193	1196	Myrtenal	---	1.4	---
1194	1195	Myrtenol	---	1.2	---
1196	1197	Methyl chavicol (=Estragol)	---	0.2	---
1198	1201	*cis*-Piperitol	---	0.8	0.1
1206	1207	Oct-3*E*-enyl acetate	---	---	0.1
1217	1218	*trans*-Carveol	---	3.8	0.2
1227	1228	*cis-p*-Mentha-1(7),8-dien-2-ol	---	0.1	---
1230	1232	*cis*-Carveol	---	1.1	0.1
1242	1242	Carvone	---	3.8	0.2
1247	1249	Linalyl acetate	tr	---	---
1266	1270	*iso*-Piperitenone	---	0.6	---
1273	1277	Perilla aldehyde	---	0.5	---
1287	1287	Limonene dioxide	---	0.7	---
1296	1299	Perilla alcohol	---	0.4	---
1303	---	Unidentified ^d^	---	1.1	---
1316	1324	Limonene hydroperoxide	---	1.1	---
1343	1346	Limonene-1,2-diol	---	2.6	---
1344	1349	7-*epi*-Silphiperfol-5-ene	---	---	0.3
1345	1349	α-Cubebene	0.2	---	---
1355	1340	*p*-Mentha-6,8-diene-2-hydroperoxide	---	1.2	---
1367	1371	α-Ylangene	tr	---	---
1374	1375	α-Copaene	4.5	---	0.1
1376	1380	Daucene	---	---	0.4
1377	1374	Isoledene	---	---	0.3
1379	1382	Modheph-2-ene	---	---	0.4
1381	1382	β-Bourbonene	tr	---	---
1385	1387	β-Cubebene	0.4	---	0.1
1386	1385	α-Isocomene	---	---	0.1
1387	1390	β-Elemene	0.3	---	0.4
1392	1394	Sativene	---	---	0.1
1398	1405	(*Z*)-Caryophyllene	0.2	---	---
1404	1406	α-Gurjunene	0.1	---	---
1408	1411	β-Isocomene	---	---	0.1
1418	1417	(*E*)-Caryophyllene	13.3	---	5.5
1427	1430	β-Copaene	0.2	---	0.2
1430	1433	*trans*-α-Bergamotene	---	---	1.1
1432	1440	6,9-Guaiadiene	---	---	0.2
1433	1436	α-Guaiene	1.8	---	---
1436	1438	Aromadendrene	0.2	---	0.1
1445	1449	(*E*)-Lachnophyllum acid	---	---	0.2
1451	1452	(*E*)-β-Farnesene	---	---	6.7
1453	1454	α-Humulene	5.4	0.3	0.7
1457	1463	*cis*-Cadina-1(6),4-diene	---	---	0.4
1460	1458	*allo*-Aromadendrene	41.2	---	---
1469	---	Unidentified ^e^	---	---	1.3
1472	1472	*trans*-Cadina-1(6),4-diene	0.5	---	0.2
1476	1479	α-Amorphene	0.1	---	---
1478	1483	Germacrene D	0.3	---	2.1
1481	1483	*trans*-β-Bergamotene	---	---	0.2
1486	1489	β-Selinene	0.5	---	---
1488	1491	Viridiflorene	0.2	---	---
1492	1497	Bicyclogermacrene	---	---	0.3
1493	1497	α-Selinene	0.3	---	---
1495	1497	α-Muurolene	0.4	---	0.1
1498	1505	α-Bulnesene	1.8	---	---
1501	1505	(*E*,*E*)-α-Farnesene	---	---	0.1
1504	1514	(*Z*)-Lachnophyllum acid	---	0.2	0.8
1507	1510	(*E*)-Lachnophyllum ester	---	---	0.4
1510	1512	γ-Cadinene	0.4	---	0.1
1515	1515	(*Z*)-Lachnophyllum ester	---	5.5	20.7
1515	1518	δ-Cadinene	0.6	---	---
1518	1519	*trans*-Calamenene	0.3	---	---
1521	1523	β-Sesquiphellandrene	---	---	0.3
1531	1532	Tridec-11-yn-1-ol	---	---	0.3
1533	1538	α-Cadinene	0.1	---	---
1538	1541	α-Calacorene	0.1	---	---
1556	1557	Germacrene B	---	---	0.1
1558	1560	(*E*)-Nerolidol	---	0.2	1.8
1559	1564	β-Calacorene	0.1	---	---
1565	1566	1,5-Epoxysalvial-4(14)-ene	---	---	0.2
1566	1568	Dendrolasin	---	---	0.1
1567	1567	Palustrol	0.1	---	---
1574	1576	Spathulenol	1.3	---	5.2
1580	1577	Caryophyllene oxide	12.2	1.1	5.8
1582	1590	Globulol	0.4	---	0.5
1589	1593	Salvial-4(14)-en-1-one	---	0.1	0.2
1590	1594	Viridiflorol	0.8	---	0.3
1593	1599	Cubeban-11-ol	0.2	---	---
1599	1601	Carotol	---	---	1.1
1601	1605	Ledol	0.6	---	---
1606	1611	Humulene epoxide II	2.2	2.9	0.4
1624	1628	1-*epi*-Cubenol	0.2	---	---
1629	1629	*iso*-Spathulenol	---	---	0.6
1633	1635	Caryophylla-4(12),8(13)-dien-5β-ol	0.2	---	---
1635	1632	Muurola-4,10(14)-dien-1β-ol	---	---	0.7
1638	1643	τ-Cadinol	0.2	---	0.4
1640	1644	τ-Muurolol	0.1	---	0.3
1643	1643	α-Muurolol	0.2	---	---
1643	1644	*allo*-Aromadendrene epoxide	---	0.3	---
1652	1655	α-Cadinol	0.6	0.3	0.4
1655	1655	Eudesma-4(15),7-dien-1α-ol	---	---	0.1
1661	1664	*cis*-Calamenen-10-ol	0.1	---	---
1666	1666	14-Hydroxy-9-*epi*-(*E*)-caryophyllene	0.1	---	---
1669	1677	Cadalene	0.1	---	---
1686	1685	Eudesma-4(15),7-dien-1β-ol	---	0.4	0.1
1698	1704	*cis*-Thujopsenol	0.1	---	---
1717	---	Unidentified ^f^	---	1.0	---
1738	1740	8α,11-Elemodiol	0.1	---	---
1751	1748	Khusimol	1.5	---	---
1790	1792	14-Hydroxy-δ-cadinene	---	---	0.2
1800	---	Unidentified ^g^	1.1	---	---
1833	1836	Neophytadiene	---	---	0.2
1857	1860	Platambin	0.1	0.5	0.1
1882	1884	Corymbolone	0.2	---	---
2103	2102	Phytol	tr	---	0.1
		Monoterpene hydrocarbons	1.5	52.7	31.8
		Oxygenated monoterpenoids	0.3	26.4	1.9
		Sesquiterpene hydrocarbons	73.7	0.3	20.7
		Oxygenated sesquiterpenoids	21.3	5.7	18.5
		Diterpenoids	trace	---	0.4
		Others	trace	7.2	22.9
		Total Identified	96.8	92.3	96.1

^a^ RI_calc_ = Retention Index calculated with respect to a homologous series of n-alkanes on a ZB-5 column. ^b^ RI_db_ = Retention Index from the databases [36,37,38,39]. ^c^ tr = trace (< 0.05%). ^d^ MS(EI): 150(3%), 135(51%), 121(29%), 119(38%), 109(42%), 107(66%), 93(97%), 91(89%), 81(50%), 79(100%), 69(82%), 67(37%), 55(65%), 53(40%), 43(75%), 41(85%). ^e^ MS(EI): 204(25%), 189(3%), 161(100%), 147(9%), 133(28%), 120(48%), 119(25%), 105(51%), 91(47%), 69(20%), 57(19%), 55(21%), 41(20%). ^f^ MS(EI): 175(3%), 135(11%), 111(48%), 93(20%), 83(19%), 67(19%), 55(26%), 43(100%), 41(20%). ^g^ MS(EI): 218(29%), 203(28%), 189(100%), 175(46%), 147(34%), 133(61%), 119(38%), 105(70%), 91(90%), 79(42%), 67(43%), 55(34%), 41(52%).

**Table 2:** Major components of *Conyza bonariensis*, *Conyza canadensis*, and *Conyza sumatrensis* essential oils from different geographical locations.

*Conyza* Species (Collection Site)	Major Components (>5%)	Ref.
*C. bonariensis* aerial parts EO (Chapada dos Guimarães, Mato Grosso, Brazil)	limonene (6.9%), (*E*)-caryophyllene (14.4%), (*E*)-β-farnesene (23.3%), germacrene D (15.3%), bicyclogermacrene (8.3%), spathulenol (7.6%)	[40]
*C. bonariensis* aerial parts EO (Melgaço, Pará, Brazil)	limonene (22.9%), (*E*)-caryophyllene (13.3%), *trans*-α-bergamotene (5.3%), (*E*)-β-farnesene (20.1%), bicyclogermacrene (6.6%), spathulenol (6.3%)	[40]
*C. bonariensis* aerial parts EO (Peixe-Boi, Pará, Brazil)	(*E*)-caryophyllene (13.3%), *trans*-α-bergamotene (8.1%), (*E*)-β-farnesene (30.9%)	[40]
*C. bonariensis* aerial parts EO (alta Floresta, Mato Grosso, Brazil)	limonene (12.6%), (*E*)-caryophyllene (13.0%), (*E*)-β-farnesene (19.1%), germacrene D (13.2%), bicyclogermacrene (6.3%), spathulenol (5.7%)	[40]
*C. bonariensis* aerial parts EO (Macapá, Amapá, Brazil)	limonene (58.4%), (*E*)-β-farnesene (7.0%)	[40]
*C. bonariensis* aerial parts EO (Rio de Janeiro, Brazil)	limonene (45.0%), (*E*)-β-ocimene (13.0%), (*E*)-β-farnesene (6.6%), germacrene D (6.4%)	[41]
*C. bonariensis* leaf EO (Minas Gerais State, Brazil)	limonene (29.6%), *trans*-α-bergamotene (10.3%), matricaria methyl ester (8.3%), β-copaen-4α-ol (7.4%)	[42]
*C. bonariensis* aerial parts EO (Athens, Greece)	limonene (8.3%), (*E*)-β-ocimene (11.5%), (*E*)-β-farnesene (8.1%), (*Z*)-lachnophyllum ester (21.2%), matricaria ester (17.5%)	[43]
*C. bonariensis* aerial parts EO (Southwestern Misiones Province, Argentina)	limonene (13.5%), (*E*)-β-ocimene (13.3%), *p*-mentha-1,3,8-triene (5.2%), germacrene D (14.6%), bicyclogermacrene (6.6%)	[44]
*C. bonariensis* leaf EO (Monastir, Tunisia)	limonene (5.8%), terpinolene (5.3%), (*E*)-β-farnesene (7.5%), matricaria ester (17.8%), caryophyllene oxide (7.8%)	[45]
*C. bonariensis* aerial parts EO (Cagliari, Sardinia, Italy)	limonene (5.1%), carvacrol (9.8%), α-curcumene (10.2%), spathulenol (18.6%), caryophyllene oxide (18.7%), neophytadiene (6.1%)	[46]
*C. bonariensis* leaf EO (Mérida State, Venezuela)	limonene (5.1%), (*Z*)-β-ocimene (5.1%), (*E*)-β-ocimene (20.7%), (*E*)-β-farnesene (37.8%), α-farnesene (5.6%), β-sesquiphellandrene (9.8%)	[47]
*C. bonariensis* leaf EO (Kabianga, Kericho, Kenya)	β-pinene (5.4%), limonene (8.3%), 2,6,7,7a-tetrahydro-1,5-dimethyl-1*H*-indene-3-carboxaldehyde (49.1%) ^a^	[48]
*C. bonariensis* aerial parts EO (Parana State, Brazil)	limonene (66.3%), 2-heptyl acetate (6.9%)	[49]
*C. bonariensis* aerial parts EO	(*E*)-caryophyllene (13.3%), α-humulene (5.4%), *allo*-aromadendrene (41.2%), caryophyllene oxide (12.2%)	this work
*C. canadensis* aerial parts EO (Plovdiv, Bulgaria)	limonene (77.7–89.4%)	[50]
*C. canadensis* aerial parts EO (Łódź, Poland)	limonene (76.3%)	[51]
*C. canadensis* aerial parts EO (Alps, France)	limonene (83.2%)	[51]
*C. canadensis* aerial parts EO (Rome, Italy)	limonene (70.3%), (*E*)-β-ocimene (5.5%)	[51]
*C. canadensis* aerial parts EO (Seville, Spain)	limonene (51.4%), (*E*)-β-ocimene (13.4%), *trans*-α-bergamotene (11.9%)	[51]
*C. canadensis* aerial parts EO (Belgium)	limonene (68.0%), (*E*)-β-ocimene (5.1%), *trans*-α-bergamotene (5.4%), germacrene D (7.3%) (*Z*,*Z*)-matricaria ester (6.1%)	[51]
*C. canadensis* aerial parts EO (Plovdiv, Bulgaria)	limonene (87.9%)	[51]
*C. canadensis* aerial parts EO (Vilnius, Lithuania)	limonene (77.7%), *trans*-α-bergamotene (5.5%)	[51]
*C. canadensis* aerial parts EO (Israel)	limonene (54.9%), (Z)-β-farnesene (6.3%) (*Z*,*Z*)-matricaria ester (7.7%)	[51]
*C. canadensis* aerial parts EO (Kerman, Iran)	myrcene (8.9%), limonene (12.3%), (*E*)-β-farnesene (14.6%), *ar*-curcumene (7.8%), zingiberene (5.5%), spathulenol (14.1%), isospathulenol (7.7%), phytol (7.3%)	[52]
*C. canadensis* aerial parts EO (Athens, Greece)	β-pinene (9.5%), limonene (57.3%), matricaria ester (14.4%)	[43]
*C. canadensis* aerial parts EO (Korea)	limonene (68.3%), (*E*)-β-ocimene (15.9%) ^b^	[53]
*C. canadensis* EO (China)	limonene (14.8%), *epi*-bicyclosesquiphellandrene (11.0%), C_7_H_30_B_4_Si (25.1%) ^c^, 1-phenyl-1-nonyne (7.3%)	[54]
*C. canadensis* aerial parts EO (Szeged, Hungary)	limonene (79.2%)	[55]
*C. canadensis* aerial parts EO (Manavgat, Antalya, Turkey)	β-pinene (9.7%), limonene (28.1%), spathulenol (16.3%)	[56]
*C. canadensis* aerial parts EO	β-pinene (8.8%), limonene (41.5%), (*Z*)-lachnophyllum ester (5.5%)	this work
*C. sumatrensis* aerial parts EO (Rondôndia state, Brazil)	sabinene (5.3%), limonene (22.9%), (*E*)-β-ocimene (5.0%), (*E*)-β-farnesene (5.3%), (*Z*)-lachnophyllum ester (43.7%)	[57]
*C. sumatrensis* leaf EO (N’gorato village, Côte d’Ivoire)	limonene (13.0%), (*E*)-β-ocimene (6.5%), (*E*)-caryophyllene (10.5%), (*E*)-β-farnesene (17.0%), (*Z*)-lachnophyllum ester (5.9%), germacrene D (13.6%), bicyclogermacrene (5.2%)	[58]
*C. sumatrensis* leaf EO (Monastir, Tunisia)	matricaria ester (7.5%), spathulenol (13.8%), caryophyllene oxide (20.5%)	[59]
*C. sumatrensis* aerial parts EO	limonene (25.5%), (*E*)-caryophyllene (5.5%), (*E*)-β-farnesene (6.7%), (*Z*)-lachnophyllum ester (20.7%), spathulenol (5.2%), caryophyllene oxide (5.8%)	this work

^a^ The identification of this compound is uncertain; it is not found in the *Dictionary of Natural Products* [60]. ^b^ This compound was listed as δ-3-carene, but the retention time is more consistent with (*E*)-β-ocimene rather than δ-3-carene. ^c^ The identification of this compound (2,3-μ-trimethylsilyl-C,C′-dimethyl-4,5-dicarba-*nido*-hexaborane) is not correct; the compound listed is not a natural product.

**Table 3:** Mosquito larvicidal activity and insecticidal activity of *Conyza* essential oils.

	**24 h**				
**Essential Oil or** **Major Compound**	**LC_50_ (95% Limits), μg/mL**	**LC_90_ (95% Limits), μg/mL**	**χ^2^**	***p***	**Slope**
	*Aedes aegypti*				
*C. bonariensis*	69.71 (64.82–75.36)	88.61 (82.13–97.54)	9.39	0.009	9.45
*C. canadensis*	9.801 (8.730–10.986)	23.27 (19.93–28.36)	8.70	0.069	12.18
*C. sumatrensis*	21.74 (20.16–23.36)	31.02 (28.29–35.50)	0.131	0.988	7.98
β-Pinene	23.63 (22.16-25.33)	32.12 (29.47-36.00)	0.225	0.994	7.69
Limonene	17.66 (16.45–18.97)	23.62 (22.03–25.73)	0.784	0.941	10.68
(*E*)-Caryophyllene	70.80 (65.49–76.69	107.2 (98.4–118.6)	4.08	0.395	12.75
α-Humulene	53.05 (48.69–58.08)	82.78 (75.81–91.87)	15.9	0.003	12.79
Caryophyllene oxide	136.6 (129.2–143.9)	180.2 (171.4–191.2)	30.1	0.000	12.37
Permethrin control	0.000643 (0.000551–0.00753)	0.00246 (0.00192–0.00344)	12.5	0.006	11.57
	*Aedes albopictus* ^a^				
*C. bonariensis*	81.13 (74.61–87.97)	127.1 (117.5–139.9)	0.395	0.821	11.44
*C. canadensis*	18.04 (16.71–19.52)	26.20 (24.22–28.82)	1.46	0.834	11.30
*C. sumatrensis*	19.13 (17.73–20.66)	27.49 (25.41–30.38)	3.19	0.364	9.97
Permethrin control	0.0024 (0.0021–0.0026)	0.0042 (0.0038–0.0049)	4.64	0.031	8.45
	*Culex quinquefasciatus*				
*C. bonariensis*	130.0 (122.5–138.8)	178.4 (165.6–197.2)	0.675	0.713	8.97
*C. canadensis*	39.37 (36.83–42.00)	52.29 (49.04–56.56)	0.493	0.974	10.49
*C. sumatrensis*	26.74 (24.80–29.20)	36.83 (33.56–41.92)	8.97	0.030	7.96
β-Pinene	30.46 (28.21–33.21)	41.58 (38.10–46.58)	0.399	0.983	9.38
Limonene	31.63 (29.37–34.50)	41.51 (38.03–46.78)	0.874	0.928	8.23
(*E*)-Caryophyllene	165.4 (157.5–174.0)	220.6 (207.8–238.5)	10.0	0.040	9.91
α-Humulene	108.3 (101.4–115.5)	158.2 (148.5–170.5)	1.0	0.910	13.32
Caryophyllene oxide	98.52 (90.70–108.68)	144.5 (129.6–165.7)	1.60	0.809	9.20
Permethrin control	0.0165 (0.0149–0.0181)	0.0305 (0.0266–0.0367)	5.24	0.073	10.12
	*Diplonychus rusticus* ^a^				
*C. canadensis*	135.7 (129.3–142.8)	182.5 (172.6–195.5)	7.78	0.051	12.35
*C. sumatrensis*	111.0 (106.1–116.7)	137.0 (129.5–147.6)	16.1	0.001	9.85
	**48 h**				
**Essential Oil or** **Major Compound**	**LC_50_ (95% Limits), μg/mL**	**LC_90_ (95% Limits), μg/mL**	**χ^2^**	***p***	**Slope**
	*Aedes aegypti*				
*C. bonariensis*	63.85 (59.07–70.75)	81.84 (74.16–94.79)	3.43	0.180	6.89
*C. canadensis*	7.091 (6.099–8.141)	22.46 (18.63–28.59)	5.98	0.201	11.63
*C. sumatrensis*	22.52 (21.18–23.87)	29.00 (27.23–31.68)	0.0488	0.997	10.12
β-Pinene	22.91 (21.29–24.85)	31.37 (29.03–35.03)	0.323	0.988	9.08
Limonene	17.43 (16.24–18.74)	23.17 (21.58–25.28)	0.664	0.956	10.48
(*E*)-Caryophyllene	65.92 (60.45–72.08)	106.4 (98.4–116.7)	14.2	0.007	13.10
α-Humulene	46.25 (42.27–50.94)	74.14 (67.47–82.99)	19.2	0.001	12.21
Caryophyllene oxide	120.2 (112.7–127.5)	165.4 (156.4–176.6)	19.8	0.001	12.34
Permethrin control	0.000575 (0.000483–0.00688)	0.00281 (0.00208–0.00423)	5.29	0.152	10.93
	*Aedes albopictus* ^a^				
*C. bonariensis*	69.42 (63.20–75.93)	113.2 (103.8–125.8)	3.10	0.212	10.72
*C. canadensis*	15.12 (13.93–16.47)	22.67 (20.84–25.09)	7.23	0.124	12.22
*C. sumatrensis*	18.43 (17.05–19.93)	26.76 (24.71–29.58)	4.25	0.236	8.44
	*Culex quinquefasciatus*				
*C. bonariensis*	108.1 (101.4–115.1)	152.1 (142.4–165.1)	2.32	0.313	10.84
*C. canadensis*	29.81 (27.33–32.68)	47.06 (43.03–52.39)	14.5	0.006	12.17
*C. sumatrensis*	22.95 (21.22-25.08)	33.06 (30.07-37.60)	2.38	0.498	9.37
β-Pinene	28.36 (26.20–31.19)	39.01 (35.41–44.50)	2.41	0.661	8.39
Limonene	29.15 (26.89–31.98)	40.83 (37.19–46.07)	7.05	0.133	9.50
(*E*)-Caryophyllene	138.5 (129.3–148.5)	215.3 (200.1–234.9)	13.5	0.009	13.11
α-Humulene	87.81 (81.14–94.89)	140.0 (130.0–152.7)	9.80	0.044	13.50
Caryophyllene oxide	95.19 (86.69–106.26)	141.0 (127.6–160.8)	4.01	0.405	10.12
	*Diplonychus rusticus* ^a^				
*C. canadensis*	124.0 (118.0–130.4)	165.0 (156.1–176.6)	1.17	0.760	12.17
*C. sumatrensis*	107.8 (103.1–113.4)	133.6 (126.1–144.4)	8.07	0.045	9.37

^a^*Aedes albopictus* and *Diplonychus rusticus* were obtained from the wild; the limited numbers of organisms available precluded screening of individual components on these two insect species.
